# Realisation of Solid-State Electrochromic Devices Based on Gel Electrolyte

**DOI:** 10.12688/f1000research.73661.2

**Published:** 2022-06-06

**Authors:** Benedict Wen-Cheun Au, Kah-Yoong Chan, Mohd Zainizan Sahdan, Abraham Shiau-Iun Chong, Dietmar Knipp

**Affiliations:** 1 Faculty of Engineering, Multimedia University, Cyberjaya, Selangor, 63100, Malaysia; 2 Faculty of Electrical and Electronic Engineering, Universiti Tun Hussein Onn Malaysia, Parit Raja, Batu Pahat, Johor, 86400, Malaysia; 3 KESPRO Consultants Sdn Bhd, KESPRO Consultants Sdn Bhd, Bandar Sunway, Subang Jaya, Selangor, 47500, Malaysia; 4 School of Engineering and Science, Jacobs University Bremen, Bremen, Bremen, 28759, Germany

**Keywords:** Electrochromic Device, Solid Polymer Electrolyte, Tungsten Oxide

## Abstract

**Background**: In the last decade, there has been much interest in the area of solid polymer electrolyte (SPE) to address the issues of electrolyte leakage and evaporation in electrochromic devices (ECD). ECD is a state-of-the-art technology having the ability to change from transparent state to opaque state under the influence of a small applied voltage for energy saving applications.

**Methods: **In this work, tungsten oxide (WO _3_) films were fabricated via the sol-gel spin-coating method. Subsequently, ECDs were assembled based on SPE and liquid polymer electrolyte (LPE), respectively using indium doped tin oxide (ITO) coated glass as conducting electrodes and WO _3_ films as working electrode.

**Results:** Cyclic voltammetry (CV) results revealed reduced ionic conductivity of conducting ions in SPE based ECD (SECD) owing to increased viscosity by addition of PMMA. However, lesser time was required for the colouration process. LPE based ECD (LECD) showed higher colouration efficiency (CE) compared to its SECD counterpart. This is attributed to its larger optical modulation.

**Conclusions:** This work presents a comparison between the performance of LECD and SECD in terms of electrochromic (EC) and optical properties. They were analysed through CV, chronoamperometry (CA) and ultraviolet-visible (UV-Vis) spectrophotometer. Furthermore, this work provides an insight on the employment of solid-state electrolytes in ECDs in view of the persistent leakage and evaporation problems in ECD implementation.

## Introduction

Electrochromism is an occurrence where the colour of electrochromic (EC) materials changes upon the application of a minute potential difference. ^1^ Since its discovery by Deb, ^2^ it has generated wide spread interest among researchers in a range of applications, including smart-windows, ^3^ rear-view mirrors ^4^ and sun roofs. ^5^ Lately, researchers have attempted solid polymer electrolytes (SPE) as an alternative to conventional liquid polymer electrolytes (LPE) as a mean to address issues such as electrolyte leakage and stability. ^6^

SPEs are made by adding a polymer material to solidify the polymer electrolyte into a solid thin film layer upon solvent evaporation. Gelatin is one of the polymer materials attempted for Li based SPEs in electrochromic devices (ECDs). ^7^ Ramadan *et al.* prepared ECD with gelatin cross-linking with formaldehyde, plasticized with glycerol and contained different LiClO _4_ concentration. ^8^ The device revealed 38% optical modulation at 600 nm wavelength with colouration efficiency (CE) of 23 cm ^2^ C ^-1^. In addition, with its good mechanics, adhesion to electrode and optical properties, it is deemed as promising for EC smart window applications. Poly (methyl-methacrylate) (PMMA) is another polymer material used in fabricating SPEs for ECDs, as it is well-known for its chemistry and lower cost of processing as laminates. ^9^ Anamika assembled a complementary ECD based on PMMA:PC:LiClO _4_ solid electrolyte leading to a ITO/WO _3_/PMMA:PC:LiClO _4_/NiO/ITO structure. This ECD demonstrated fast colouring and bleaching time of 1.2 s and 1.5 s respectively, with a large optical modulation of 50.3% at 630 nm wavelength. On top of that, CE was calculated to be as high as 243 cm ^2^ C ^-1^. ^10^ In another work, Evecan and Zayim produced an all-solid-state ECD using PMMA:PC:LiClO _4_ as solid electrolyte which had respond times of approximately 10 s. Besides that, the large optical modulation of 48.07% led to a high CE of 68.7 cm ^2^ C ^-1^. ^11^

In this work, a comparison between LPE and SPE based ECDs is made in terms of EC and optical properties. Propylene carbonate (PC):Lithium perchlorate (LiClO _4_) was used as LPE, while PC:LiClO _4_:PMMA was used as SPE in ECD study. Their respective optical and EC properties are elucidated and discussed in this paper.

## Methods

This section describes the experimental design. The EC WO _3_ films here were produced according to the sol-gel spin-coated method in our previous work. ^12^ Tungsten hexachloride (WCl _6_) powder was adopted as precursor for WO _3_, absolute ethanol (C _2_H _6_O) was used as solvent, glacial acetic acid (CH _3_COOH) was used as chelating agent and hydrogen peroxide (H _2_O _2_) was used as strong oxidizing agent. 1 g of WCl _6_ powder and 2 ml of glacial acetic acid were added to 20 ml absolute ethanol. The mixture was stirred for 30 minutes before adding 2 ml of H _2_O _2_ and subsequently stirred for another 30 minutes at room temperature. Next, the WO _3_ solution was continuously stirred for two hours at 40 °C to obtain a homogenous clear solution. Preceding the deposition of WO _3_ films, acetone and IPA were used to wash the ITO coated glass substrates for 10 minutes, respectively. Then, the sol-gel solution was shifted to the ITO coated glass substrates and spun for 30 seconds at 3000 RPM. Subsequently, the fresh WO _3_ coating was heated at 100 °C on a hotplate for three minutes to allow solvent vaporisation. Multiple coatings were accumulated to obtain a film thickness of approximately 338 nm. Lastly, the coated WO _3_ films were heat-treated at 250 °C for one hour.

7 g of PMMA powder was added to 1 M PC:LiClO _4_ polymer electrolyte and stirred for the formation of SPE. Subsequently, a solid-state ECD was assembled with PC:LiClO _4_:PMMA SPE sandwiched between WO _3_ film as the colour changing layer and ITO coated glass as the counter electrode, forming a ITO/WO _3_/PC:LiClO _4_:PMMA/ITO device structure. An acrylic frame of 1 mm thickness was used as spacer to store the SPE between the electrodes and the boundaries of the ECD were closed up with silicone sealant to avert moisture from entering the ECD. Identical to the solid-state ECD, 1 M PC:LiClO _4_ liquid polymer electrolyte (LPE) was used for the non-solid-state ECD, forming a ITO/WO _3_/PC:LiClO _4_/ITO device structure. Both the sealed solid-state and non-solid state ECDs were dried overnight before measurements were carried out. For simplicity purposes, the SPE based ECD is named as SECD, while the LPE based ECD is named as LECD in this paper.

Optical properties of the ECDs were evaluated by ultraviolet-visible (UV-Vis) spectrophotometer. EC properties were measured using a two-electrode setup via a potentiostat-galvanostat. The anodic and cathodic peak currents and diffusion coefficient for the insertion and extraction of Li ^+^ ions were analyzed by performing the cyclic voltammetry (CV) measurements between −3.0 V to 3.0 V at a scan rate of 0.1 V s ^-1^. Chronoamperometry (CA) assessments were conducted to analyse the switching kinetics of the ECDs while documenting the transmittances in the dark blue and translucent states in the range of 280 nm to 900 nm wavelength.

## Results

**Figure 1.**
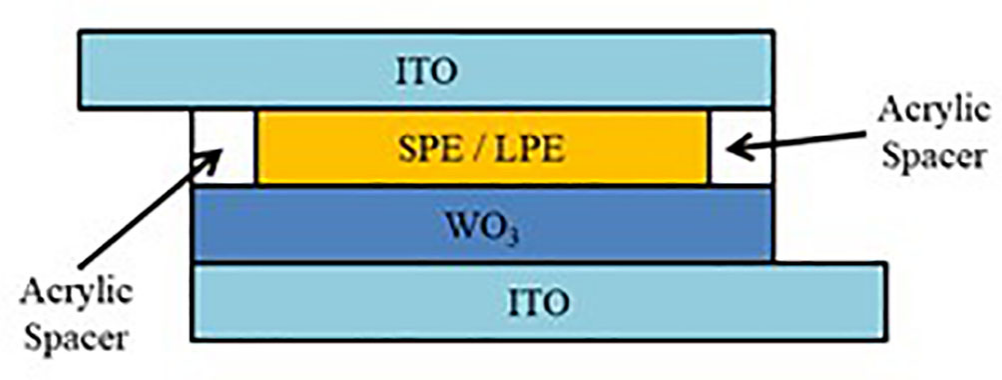
Device structure of SECD and LECD.

The device structure for both SECD and LECD is presented in Figure 1. Sol-gel deposited WO _3_ films are used as the EC layer and ITO coated glasses are used as the conductive electrodes. The SPE and LPE are sandwiched in between the ECD using an acrylic spacer. To explain further, the WO _3_ films are the colour changing layer, ITO coated glasses conduct electricity across the ECDs and the SPE and LPE supplies the necessary ions for ECD operation.

**Figure 2.**
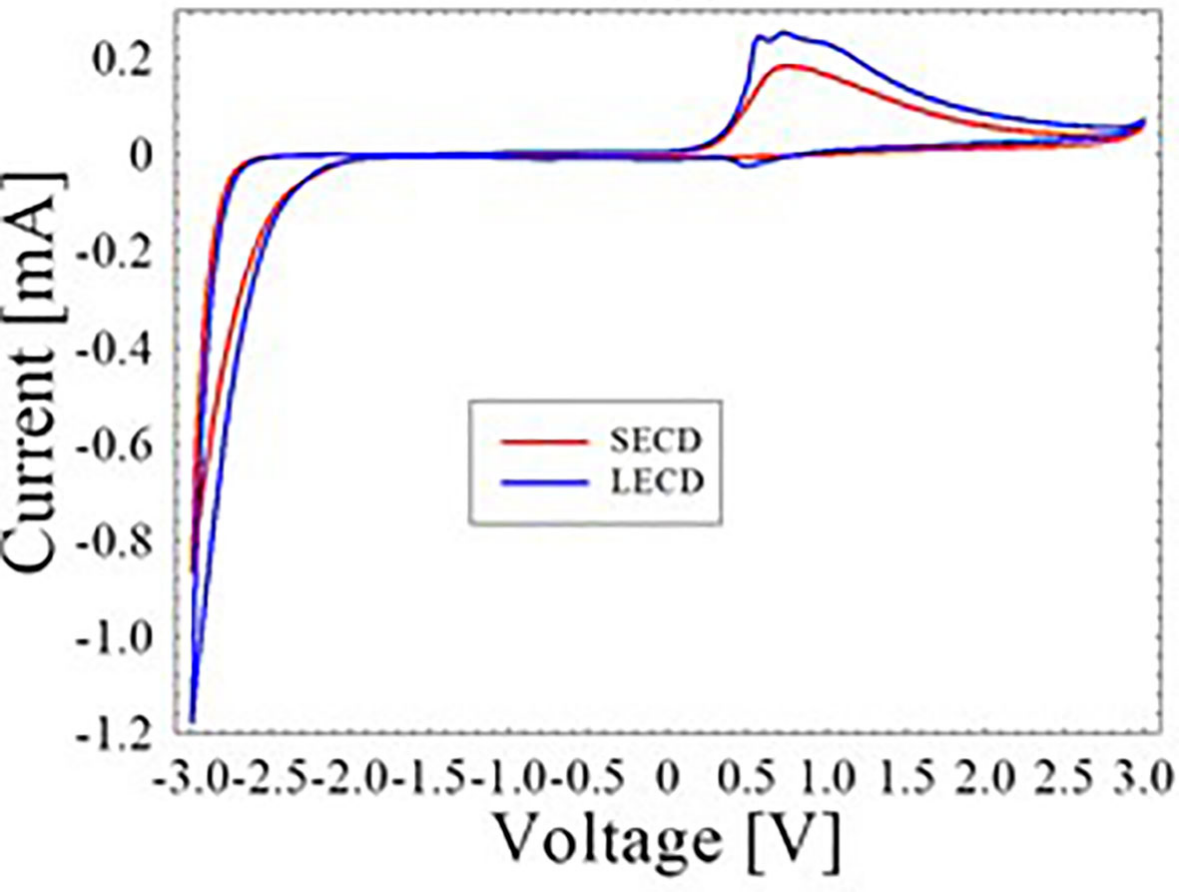
CV curves of SECD and LECD.

CV characteristics of the SECD and LECD are depicted in Figure 2. Upon applying −3.0 V, both the ECDs changed into its dark blue state. This is attributed to the transition from the W ^6+^ state to the W ^5+^ state of the WO _3_. On the other hand, the ECDs returned to its transparent state when 3.0 V was applied. ^13^ To explain further, ions were inserted into the WO _3_ layer during the negative voltage cycle and hence its dark blue appearance. In the positive voltage cycle, extracted ions meaning the WO _3_ layer became transparent again. This reversible reaction can be represented by the chemical equation below ^14^:

$${\mathrm{WO}}_{3}\;\left(\text{colourless}\right)+x{\mathrm{Li}}^{+}+xe^{-}\leftarrow \to \;{\mathrm{Li}}_{x}{\mathrm{WO}}_{3}\;\left(\text{dark blue}\right)$$

where Li ^+^ is the ions in the SPE and LPE, *x* is the ion concentration. In Figure 2, the area under the CV curve of LECD is larger compared to the SECD which indicate greater charge storage capacity. ^15^

The LECD anodic peak current of 0.253 mA was found to be greater than the 0.184 mA of SECD. Besides, the LECD cathodic peak current of −1.177 mA was also observed to be greater than the −0.865 mA of SECD. These results were further used to compute the diffusion coefficient during insertion and extraction procedure of Li ^+^ ions through the Randles-Sevcik equation ^16^:

$$i_{p}=2.72\times 10^{5}\times n^{3/2}\times D^{1/2}\times C_{o}\times \upsilon^{1/2}$$

where i _p_ is the anodic peak current and cathodic peak current respectively, n is the number of electrons, D is the diffusion coefficient, C _o_ is the concentration of electrolyte and υ is the scan rate. Subsequently, the mentioned parameter above is summarized in Table 1. Both the anodic diffusion coefficient of 6.168 × 10 ^−13^ cm ^2^ s ^−1^ and cathodic diffusion coefficient of 1.333 × 10 ^−11^ cm ^2^ for LECD were computed to be greater than the 3.261 × 10 ^−13^ cm ^2^ s ^−1^ and 7.189 × 10 ^−12^ cm ^2^ s ^−1^ of SECD, respectively. Deepa *et al.* explained that the addition of PMMA in the polymer electrolytes increases the viscosity tremendously, leading to a decrease in conductivity. ^17^ Therefore, the lower diffusion coefficient in SECD is attributable to the increase in viscosity. However, it is worth noting that even though SPE in SECD is highly viscous, the anodic and cathodic diffusion coefficients compared to LECD merely differs by a factor of 2, which is only a slight decrease. The results here are comparable to the work of Kufian *et al.* ^18^ and Wu *et al.* ^9^

**Table 1.** Diffusion coefficient of the Li ^+^ ions in SECD and LECD.

Type of ECD	Anodic peak current (mA)	Cathodic peak current (mA)	Anodic diffusion coefficient (cm ^2^/s ^-1^)	Cathodic diffusion coefficient (cm ^2^/s ^-1^)
SECD	0.1841	-0.8648	3.261 × 10 ^-13^	7.189 × 10 ^-12^
LECD	0.2533	-1.1775	6.168 × 10 ^-13^	1.333 × 10 ^-11^

CA assessments were conducted to further analyze the electrochemical behaviour of both devices. It was performed with a sweeping voltage of −3 V to 3 V for 60 s while documenting the transmittances in the dark blue and translucent states at 633 nm wavelength, simultaneously. Besides, the switching time for both the ECDs were extracted from the CA results and tabulated in Table 2.

**Table 2.** Switching time of LECD and SECD.

	Colouring time (s)	Bleaching time (s)
LECD	2.75	1.41
SECD	1.88	1.42

Switching time represents the time taken to arrive at its steady state. As shown in Table 2, LECD required more time for colouration compared to its SECD counterpart. We speculate that this is due to the difference in Li ^+^ ions in both LPE and SPE during the colouring process. As explained earlier, the solvation of Li ^+^ ions in PMMA leads to a decrease in the concentration of colouration ions. Consequently, the SECD reaches its steady state faster than the LECD which has higher concentration of colouration ions. It is worth noting that regardless of LPE or SPE, time taken for colouration is longer than decolouration. In the dark blue state, EC WO _3_ is very conductive while it exhibits insulating nature in the translucent state. This is an indication that transiting from conductive state to insulating states is a much quicker process. ^19^

**Figure 3.**
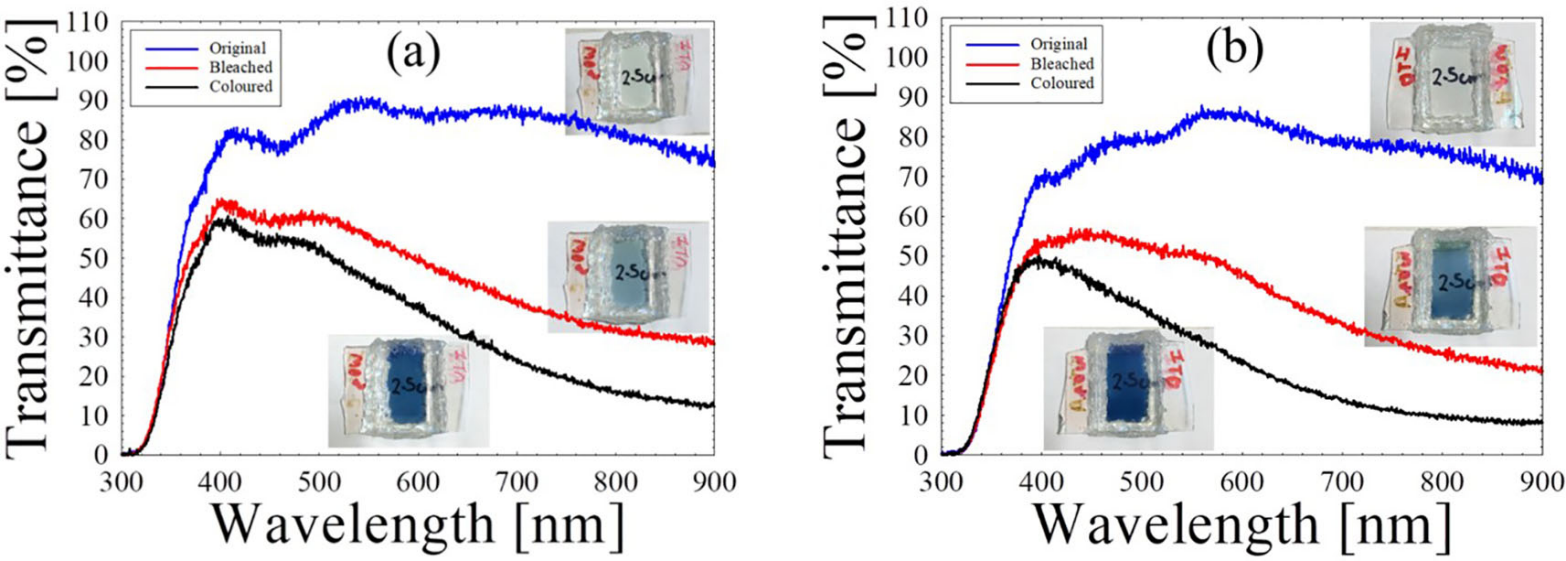
Original, bleached and coloured transmittances of (a) SECD and (b) LECD.

In terms of optical properties, the original, coloured and bleached transmittance of SECD and LECD are recorded in Figure 3(a) and 3(b) respectively, under wavelengths between 280 nm and 900 nm as well as its corresponding colour change of the physical ECDs.

Both ECDs are highly transparent with an approximate 85% in the visible range prior to any measurements. For SECD, the dark blue state exhibited 30% transmittance and the translucent state exhibited 46% with an optical modulation of 16%. On the other hand, LECD had 19% in the coloured state and 40% in the bleached state, which are lower than the SECD. However, the obtained optical modulation of 21% was slightly higher. Bohnke *et al.* explained that PMMA molecules may solvate the Li ^+^ ions via their negatively charged carbonyl group. This leads to a decrease in the overall amount of Li ^+^ ions needed for colouration process in the SPE. ^20^ Therefore, it reflects on the higher transmittance level of SECD in the coloured state compared to LECD. In the work of Namrata and Awalendra, a systematic shift of the PMMA main XRD peak to a higher angle proved the interaction between LiClO _4_ and PMMA. Consequently, this resulted in the emergence of possible complexations of Li ^+^ ions with electron abundant sites in the host polymer matrix. ^21^

Results from the optical properties were subsequently utilized to evaluate the colouration efficiency (CE) with the following equation ^22^:

$$\mathrm{CE}=\frac{\Delta \mathrm{OD}}{Q_{\text{in}}}=\frac{\log\left(\frac{T_{b}}{T_{c}}\right)}{Q_{\text{in}}}$$

where ΔOD is the change in optical density at 633 nm wavelength, and *Q*_in_ is the inserted charge. CE is a key aspect in the study of EC and is described as the ratio between optical density change and a unit of injected charge per unit area during colouration process. The CE for LECD and SECD was calculated to be 78.7 cm ^2^ C ^-1^ and 56.1 cm ^2^ C ^-1^, respectively. Despite having a greater colouring charge density of 4.11 mCcm ^-2^ in LECD compared to 3.31 mCcm ^-2^ in SECD, the better CE was mainly attributed to the larger optical modulation.

## Conclusions

ECDs based on LPE and SPE were produced using ITO coated glasses as a pair of conductive electrodes. Their respective EC properties were discussed and elucidated. CV results showed greater diffusion coefficient in LECD owing to its less viscous nature of the LPE. More time was needed for the colouration process in LECD due to the higher concentration of Li ^+^ ions. Both LECD and SECD had high transparency of approximately 85% in the original state in the visible range. Greater optical modulation was observed in LECD compared to SECD leading to greater CE. This research demonstrated that while possessing inferior EC properties, the implementation of SECDs is the way forward towards a new generation of ECDs where the persistent electrolyte leakage and evaporation problem in conventional LECDs can finally be eliminated.

## Author contributions

**Benedict Wen-Cheun Au:** Data Curation, Formal Analysis, Investigation, Methodology, Visualization, Writing – Original Draft Preparation.

**Kah-Yoong Chan:** Conceptualization, Formal Analysis, Funding Acquisition, Methodology, Project Administration, Resources, Supervision, Validation, Writing – Review & Editing.

**Mohd Zainizan Sahdan**: Validation

**Abraham Shiau-Iun Chong**: Conceptualization

**Dietmar Knipp**: Conceptualization, Validation

## Data availability

Underlying data will be shared upon request.

Dryad, https://doi.org/10.5061/dryad.qfttdz0jx

Data are available under the terms of the CC0 1.0 Universal (CC0 1.0) Public Domain Dedication.

## Ethical approval number

EA2092021
