# Supplementary material for: Realisation of Solid-State Electrochromic Devices Based on Gel Electrolyte
Source: F1000Res. 2022 Jun 6;11:380. Originally published 2022 Mar 31. [Version 2] doi: 10.12688/f1000research.73661.2 (PMC9184923; doi:10.12688/f1000research.73661.2)
Supplement: Peer review history

# Peer review: Reviewer response for version 2

**DOI:** 10.5256/f1000research.134098.r139748

**Published:** 2022-06-09

**Authors:** Mahesh A. Shinde

**Affiliations:** 1 Department of Materials Science and Engineering, and Organic and Carbon Electronics Laboratories (ORaCEL), North Carolina State University, Raleigh, NC, 27695, USA

The article proposed the Solid polymer electrolyte (SPE) for the replacement of liquid polymer electrolyte (LPE). The implementation of SPE based electrochromic devices (ECD) showed good electrochromic performance then LPE based ECD. The results appear to be compelling and warrant indexing. The manuscript contains interesting results for the field of electrochromic devices. I am satisfied with the revised version of the article.

Is the work clearly and accurately presented and does it cite the current literature?

Yes

If applicable, is the statistical analysis and its interpretation appropriate?

Yes

Are all the source data underlying the results available to ensure full reproducibility?

Partly

Is the study design appropriate and is the work technically sound?

Yes

Are the conclusions drawn adequately supported by the results?

Yes

Are sufficient details of methods and analysis provided to allow replication by others?

Partly

Reviewer Expertise:

NA

I confirm that I have read this submission and believe that I have an appropriate level of expertise to confirm that it is of an acceptable scientific standard.

# Peer review: Reviewer response for version 1

**DOI:** 10.5256/f1000research.77327.r129458

**Published:** 2022-04-25

**Authors:** Mahesh A. Shinde

**Affiliations:** 1 Department of Materials Science and Engineering, and Organic and Carbon Electronics Laboratories (ORaCEL), North Carolina State University, Raleigh, NC, 27695, USA

The article proposed the Solid polymer electrolyte (SPE) for the replacement of liquid polymer electrolyte (LPE). the implementation of SPE based electrochromic devices (ECD) showing good electrochromic performance then LPE based ECD. The results appear to be compelling and warrant indexing. The manuscript contains interesting results for the field of electrochromic devices, but some explanations are not clearly written and experimental data are not sufficient to discuss the results in detail. I feel the manuscript could be improved when properly addressing the points below.

1. The thickness of WO3 film should be mentioned in the manuscript.
2. The electrolyte composition should be explained in the manuscript such as molar ratio and weight percentage.
3. SPE electrolyte results should be compared with previous literature in tabular form.
4. Why did authors measure the electrochromic performance at 633 nm as many previous WO3 based ECD report measure electrochromic performance at 700nm wavelength?

Is the work clearly and accurately presented and does it cite the current literature?

Yes

If applicable, is the statistical analysis and its interpretation appropriate?

Yes

Are all the source data underlying the results available to ensure full reproducibility?

Partly

Is the study design appropriate and is the work technically sound?

Yes

Are the conclusions drawn adequately supported by the results?

Yes

Are sufficient details of methods and analysis provided to allow replication by others?

Partly

Reviewer Expertise:

Electrochromic devices

I confirm that I have read this submission and believe that I have an appropriate level of expertise to confirm that it is of an acceptable scientific standard, however I have significant reservations, as outlined above.

# Response

**Published:** 2022-05-13

**Authors:** Kah-Yoong Chan

**Affiliations:** Multimedia University, Malaysia

Dear Prof. Dr. Mahesh A. Shinde,

 Thank you for your comments on our manuscript titled “Realisation of Solid-State Electrochromic Devices based on Gel Electrolyte”. We have carefully reviewed the comments and have revised the manuscript accordingly. Changes to the manuscript are highlighted in red.

**Comment:**

 1. The thickness of WO _3 _film should be mentioned in the manuscript.

 Answer: The thickness is approximately of 340 nm and has been added into the manuscript.

 2. The electrolyte composition should be explained in the manuscript such as molar ratio and weight percentage.

 Answer: The electrolyte composition is added to the manuscript.

 3. SPE electrolyte results should be compared with previous literature in tabular form.

 Answer: This summary is in a Review Paper, accepted and to be published in *Polymers (Publisher: MDPI)*.

 4. Why did authors measure the electrochromic performance at 633 nm as many previous WO _3 _based ECD report measure electrochromic performance at 700 nm wavelength?

 Answer: The work here focuses on visible wavelengths ranging from 350 nm to 650 nm where 700 nm is approaching the infrared region. Besides that, this work is with reference to many existing reports which measures electrochromic properties at 633 nm.

# Peer review: Reviewer response for version 1

**DOI:** 10.5256/f1000research.77327.r129461

**Published:** 2022-04-19

**Authors:** Soo Kien Chen

**Affiliations:** 1 " Department of Physics, Faculty of Science", Universiti Putra Malaysia, Serdang, Malaysia

In the present communication, the authors evaluated and compared the electrochemical and optical properties of SECD and LECD. It was found that the SECD showed lower diffusion coefficient due to its higher viscosity. Nevertheless, its colouring time is shorter than that of the LECD.

 Comment:

 1. Method:

- “Multiple coatings were accumulated to obtain a desired film thickness.” What is the thickness of the film?
- EC properties were measured using “in” a two-electrode setup via a potentiostat-galvanostat. Reconsider "in".

2. Results and discussion

- P. 4: “Both the anodic diffusion coefficient of 3.261 1013 cm2 s -1 and cathodic diffusion coefficient of 7.189 1012 cm2 s -1 for LECD were computed to be greater than the 6.168 10-13 cm2 s -1 and 1.333 10-11 cm2 s -1 of SECD, respectively.” This behaviour is opposite to that given in Table 1.
- P5 - We speculate that this is due to the difference in Li+ ions in both LPE and SPE during “in” the colouring process. Reconsider "in".

Is the work clearly and accurately presented and does it cite the current literature?

Yes

If applicable, is the statistical analysis and its interpretation appropriate?

Not applicable

Are all the source data underlying the results available to ensure full reproducibility?

Yes

Is the study design appropriate and is the work technically sound?

Yes

Are the conclusions drawn adequately supported by the results?

Yes

Are sufficient details of methods and analysis provided to allow replication by others?

Yes

Reviewer Expertise:

Nanomaterials, magnetism

I confirm that I have read this submission and believe that I have an appropriate level of expertise to confirm that it is of an acceptable scientific standard.

# Response

**Published:** 2022-05-13

**Authors:** Kah-Yoong Chan

**Affiliations:** Multimedia University, Malaysia

Dear Prof. Dr. Soo,

 Thank you very much for your comments on our manuscript entitled “Realisation of Solid-State Electrochromic Devices based on Gel Electrolyte”. We have carefully reviewed the comments and have revised the manuscript accordingly. Changes to the manuscript are highlighted in red.

**Comment**

**Method**:

 1. “Multiple coatings were accumulated to obtain a desired film thickness.” What is the thickness of the film?

 Answer: The film thickness is approximately 340 nm.

 2.EC properties were measured using “in” a two-electrode setup via a potentiostat-galvanostat. Reconsider “in”.

 Answer: “In” has been removed from the mentioned sentence

**Results and discussion**

 P. 4: “Both the anodic diffusion coefficient of 3.261 10 ^-13^ cm ^2^ s ^-1^ and cathodic diffusion coefficient of 7.189 10 ^-12^ cm ^2^ s ^-1^ for LECD were computed to be greater than the 6.168 10 ^-13^ cm ^2^ s ^-1^ and 1.333 10 ^-11^ cm ^2^ s ^-1^ of SECD, respectively.” This behaviour is opposite to that given in Table 1.

 Answer: This is amended in the manuscript. The diffusion coefficients should be the other way round.

 2. P-5 - We speculate that this is due to the difference in Li+ ions in both LPE and SPE

                   during “in” the colouring process. Reconsider "in".

 Answer: “In” has been removed from the mentioned sentence.
